# Troubleshooting Real-Time PCR Screening for *Leishmania infantum*

**Published:** 2018-12-25

**Authors:** Philippe Gil de Mendonça

**Affiliations:** Institute of Comparative Tropical Medicine and Parasitology, Ludwig Maximilian University, Munich, Germany

## Dear Editor-in-Chief

The parasite *Leishmania infantum* is a widespread cause of concern to both human and veterinary medicine (1–3). A simple and quick real-time PCR protocol was proposed for screening samples for *L. infantum* (4). This protocol was efficiently applied in various studies (4–7). However, during informal discussions at international meetings, colleagues complained of difficulties in applying this protocol. Such complaints are common and usually overcome through optimization, however, the opportunity to use a different type of thermocycler whilst on a visit abroad flagged the seriousness of that complaint.

The reason for the difficulties encountered lies in a weakness of this PCR protocol. Indeed, the annealing temperature of the probe is substantially lower than the annealing temperature of the primers, whereas this should be the reverse. The implication of this is that if using a fast cooling thermocycler with Peltier element, the real-time PCR will work as expected ( 5), although the curves sometimes look somewhat “stretched”, which is a symptom of suboptimal PCR conditions. However, if using a slow cooling (e.g. air-cooled) thermocycler, the real-time PCR may look like it failed, as no curves will appear. In such a case, the primers annealed much earlier than the probe, and the Taq polymerase was fast enough to elongate the DNA target before the probe had attached. This results in no fluorophore release and thus no positive signal detection. However, an agarose gel electrophoresis will reveal that the PCR amplification was successful, while the fluorogenic detection failed.

This is an important limitation to be aware of. The implication is that researchers using a slow cooling thermocycler will have no chance to observe a real-time detection signal. They will have no other option than to run an agarose gel electrophoresis, thus losing both the specificity of the TaqMan probe and the sensitivity of the real-time detection. Researchers having a fast cooling thermocycler may continue using this relatively old protocol, whereas laboratories relying on air-cooled equipment ought to opt for alternative real-time PCR protocols. Many real-time PCR protocols (including TaqMan protocols) have been developed and can be tested and selected to suit the technical requirements and limitations of each laboratory. Air-cooled, i.e. slow cooling thermocyclers, are however a limiting factor in many cases! If real-time detection is unsuccessful, then sequencing of amplicons might be required to confirm specific amplification. Indeed, the added specificity of the TaqMan probe is missing in such cases. Researchers in Asia (8) cautiously opted for sequencing their *Leishmania* isolates following conventional amplification. A wise example to follow is such cases indeed.
